# PcNPF2.7 from the Xerophyte *Pugionium cornutum* Facilitates Root-to-Shoot NO_3_^−^ Transport and Affects Na^+^ Transport Under Salt Stress

**DOI:** 10.3390/biology14111590

**Published:** 2025-11-14

**Authors:** Peng-Fei Ren, Fang-Zhen Wang, Zhuo-Ma Deji, Hao-Ran Liao, Mei-Mei Cai, Ke-Yan He, Li Li, Xiao-Xing Wei, Qing Ma

**Affiliations:** 1 State Key Laboratory of Herbage Improvement and Grassland Agro-Ecosystems, College of Pastoral Agriculture Science and Technology, Lanzhou University, Lanzhou 730020, China; renpf2023@lzu.edu.cn (P.-F.R.); wangfz201809@163.com (F.-Z.W.); 220220901040@lzu.edu.cn (Z.-M.D.); liaohr2025@lzu.edu.cn (H.-R.L.); cmm3190@163.com (M.-M.C.); 2 Qinghai Academy of Animal Science and Veterinary Medicine, Qinghai University, Xining 810016, China; Keyan1961006779@163.com; 3 Xinjiang Uygur Autonomous Region Academy of Animal Science, Urumqi 830000, China; xjndlili@126.com

**Keywords:** xerophyte, *Pugionium cornutum*, *PcNPF2.7*, NO_3_^−^ transport, Na^+^ transport, salt tolerance

## Abstract

Nitrate Excretion Transporter 1 (NAXT1/NPF2.7) is known to regulate NO_3_^−^ transport in Arabidopsis, a salt-sensitive glycophyte that exhibits a significant reduction in the NO_3_^−^ content under salt stress. However, its role in the NO_3_^−^ homeostasis and salt tolerance of xerophytes, which exhibit strong stress tolerance, remains unclear. In the present study, we cloned the *NPF2.7* homolog (*PcNPF2.7*) from the xerophyte *Pugionium cornutum*, which exhibits stable NO_3_^−^ content in the shoot under salt stress, and investigated its function in ion homeostasis and salt tolerance. *PcNPF2.7* was specifically expressed in the stele tissue of roots and localized to the plasma membrane; its expression level in the roots was significantly induced by NaCl and NaNO_3_ treatments. *PcNPF2.7* overexpression driven by a stelar-specific promoter significantly increased NO_3_^−^ accumulation and reduced Na^+^ levels in the shoots of Arabidopsis under 75 mM NaCl or NaNO_3_ treatments, resulting in an enhanced salt tolerance. Furthermore, *PcNPF2.7* overexpression significantly induced *AtHKT1;1*, which mediates the unloading of Na^+^ from xylem in the roots. Taken together, our findings showed that PcNPF2.7 facilitates the transport of NO_3_^−^ from the roots to the shoots and indirectly reduces Na^+^ accumulation in the shoot, therefore contributing to the salt tolerance in plants.

## Simple Summary

*Pugionium cornutum* is a typical xerophyte indigenous to arid and semi-arid regions of northwest China, exhibiting remarkable adaptability to drought and salt stresses. Unlike salt-sensitive plants, maintaining a stable NO_3_^−^ concentration in the shoots is an important physiological strategy underlying the salt stress adaptation of *P. cornutum*. This study aimed to investigate the role of PcNPF2.7 from *P. cornutum* in the NO_3_^−^ homeostasis and salt tolerance. Our results demonstrated that *PcNPF2.7*, which was mainly expressed in the stele tissue of the roots of *P. cornutum*, facilitates root-to-shoot NO_3_^−^ transport and modulates Na^+^ accumulation in the shoot, thus contributing to the salt tolerance of the plant. The findings provide a potential candidate gene for improving salt tolerance in crops.

## 1. Introduction

Soil salinization is a major abiotic stress that inhibits crop growth and development, posing a serious threat to global food security [1]. Developing stress-tolerant crops through genetic approaches would constitute a sustainable solution for ensuring global food security [2,3]. The exploration of salt-tolerant genetic resources serves as a crucial prerequisite for such breeding practices [3,4]. Given the limited genetic potential of salt tolerance in traditional crops, extremophile plants such as xerophytes that can thrive in harsh environments represent a crucial genetic reservoir that can be utilized to enhance salt tolerance in crops [5,6,7]. Therefore, identifying and utilizing the salt tolerance genes of these plants is critical for enhancing the crop salt tolerance [8,9,10].

The perennial herb *Pugionium cornutum* L. Gaertn, belonging to the Brassicaceae family, is a typical xerophyte indigenous to the arid and semi-arid regions of northwest China [11,12,13,14,15]. This species exhibits remarkable adaptability to drought and salt stresses [11,12,13,14,15]. The crucial role of *P. cornutum* in water and soil conservation, as well as its utilization as a vegetable, forage, and traditional Chinese medicinal herb, renders it a crucial ecological barrier and resource plant [16,17]. Previous studies demonstrated that *P. cornutum* is a typical chloride (Cl^−^)-tolerant species, as it possesses a strong ability to efficiently absorb and accumulate significant quantities of Cl^−^ in the shoot for osmotic adjustment [12,13,14,18]. The enhanced Cl^−^ uptake and accumulation in most salt-sensitive plants has been shown to be commonly accompanied by a significant reduction in NO_3_^−^ accumulation under salt stress, which, in turn, inhibits the growth of these plants under saline conditions [19,20]. Unlike most salt-sensitive species, *P. cornutum* can maintain stable NO_3_^−^ concentrations in shoots when accumulating large amounts of Cl^−^ for osmotic adjustment under high salt conditions, indicating its strong capacity to maintain shoot NO_3_^−^ homeostasis in the presence of high Cl^−^ accumulation [12]. However, the molecular mechanisms underlying the maintenance of NO_3_^−^ homeostasis in *P. cornutum* under salt stress are poorly understood.

Researchers have identified a series of transporters or channels involved in NO_3_^−^ transport in plants [21,22,23]. Nitrate Transporter 1/Peptide Transporter Family (NRT1/PTR Family or NPF) has been defined, based on sequence homology and substrate specificity, as NO_3_^−^ transporters or peptide transporters [24,25]. The model plant Arabidopsis harbors 53 NPF members, including the Nitrate Excretion Transporters (NAXT) subclass members [24,25]. The genes encoding the members of this subclass are tandemly distributed on chromosome 3 and have been proposed to have evolved through gene duplication after speciation events [26]. The NAXT subclass is named after the identification of its first member, NAXT1 (subsequently named NPF2.7), and comprises seven members (named AtNPF2.1~AtNPF2.7) in Arabidopsis. Among them, the functional characteristics of three members, AtNPF2.1, AtNPF2.2, and AtNPF2.6, have not yet been identified. Among the remaining four members, AtNPF2.7 and AtNPF2.3 are reportedly involved in NO_3_^−^ efflux from the root system and long-distance transport of NO_3_^−^ from the roots to the shoots, respectively [27,28]. In addition, AtNPF2.5 and AtNPF2.4 are involved in Cl^−^ efflux from the root system and long-distance transport of Cl^−^ from the roots to the shoots, respectively [29,30]. However, current studies on the functions of NAXT subclass members have mainly focused on Arabidopsis, which exhibits a significant reduction in NO_3_^−^ concentration under salt stress. However, the role of the NAXT members in the NO_3_^−^ homeostasis and salt tolerance in *P. cornutum*, which exhibits extremely strong salt tolerance and can maintain stable NO_3_^−^ levels under salt stress, remains unclear.

In the present study, we isolated the *NPF2.7* homolog (*PcNPF2.7*) from *P. cornutum*, and analyzed its expression patterns, tissue and subcellular localization, and then investigated its role in the salt tolerance and ion accumulation characteristics after expressing it into Arabidopsis.

## 2. Materials and Methods

### 2.1. Isolation of PcNPF2.7 from P. cornutum

After germination at 28 °C for 5 d, uniform *P. cornutum* seedlings were transferred to plastic pots (6 cm × 6 cm × 5 cm) filled with coarse-grained silica sand, with one seedling per pot. The seedlings were irrigated with 1/2 Hoagland nutrient solution (containing 2 mM KNO_3_, 0.5 mM KH_2_PO_4_, 0.5 mM MgSO_4_, 0.5 mM Ca(NO_3_)_2_, 60 μM Fe-citrate, 50 μM H_3_BO_3_, 10 μM MnCl_2_, 1.6 μM ZnSO_4_, 0.6 μM CuSO_4_, and 0.05 μM Na_2_MoO_4_, pH = 5.7), and cultivated in a greenhouse as described previously [18]. After 4 weeks, the total RNA was isolated from the root tissues using a RNAprep Pure Plant Kit (TaKaRa, Dalian, China). Then the reverse transcription was performed using a SMART RACE cDNA Kit (Clontech, Mountain View, CA, USA) to create 5′- and 3′-RACE cDNA template. Based on the specific fragment of *PcNPF2.7* obtained from the transcriptomic data of *P. cornutum* [13], the 5′-end cDNA and 3′-end cDNA fragments were cloned with the nested PCR method [18]. The primer pairs for the nested amplification of the 5′-end cDNA were P1/P2 and P3/P4 (Table S1), and those for the nested amplification of 3′-end cDNA were P2/P5 and P4/P6 (Table S1). These three fragments were assembled to obtain the full-length cDNA sequence of *PcNPF2.7*.

### 2.2. Bioinformatics Analysis

Multiple alignments were conducted with other NAXT proteins in Brassicaceae plants using the DNAMAN (v6.0) software (Lynnon Biosoft, San Ramon, CA, USA). The phylogenetic tree was generated by the MEGA (v7.0) software (Premier Biosoft International, Palo Alto, CA, USA) using the maximum-likelihood method and 1000 bootstrap replicates. The transmembrane-domain prediction was performed with TMHMM Server v2.0 (http://www.cbs.dtu.dk/services/TMHMM/) (accessed on 10 December 2024).

### 2.3. Quantitative Real-Time PCR (qRT-PCR) of PcNPF2.7 in Response to NaCl and NaNO_3_ Treatments

To investigate the response of *PcNPF2.7* in *P. cornutum* to salt treatments, 4-week-old seedlings were exposed to 1/2 Hoagland solution supplemented with either 25 mM or 50 mM NaCl (Cl^−^ stress) and 25 mM or 50 mM NaNO_3_ (NO_3_^−^ stress). Roots and shoots were harvested at 0, 3, 6, 12, 24, and 48 h after applying the respective NaCl or NaNO_3_ treatments. Total RNA in these samples was extracted, and cDNA was synthesized by reverse transcription using PrimeScript^™^ RT master mix (Perfect Real Time; TaKaRa, Dalian, China). Subsequently, qRT-PCR was performed by a StepOnePlus Real-Time PCR Thermocycler (PRISM 7500, Applied Biosystems, Foster City, CA, USA). *PcACTIN2* [15] was used as the reference gene for RNA normalization. TB Green^TM^ Premix Ex Taq^TM^ mix (Takara, Dalian, China) and the specific primer pairs for *PcACTIN2* and *PcNPF2.7* (P7/P8 and P9/P10, respectively; Table S1) were used for PCR. Finally, the relative expression level of *PcNPF2.7* was calculated using the 2^−ΔΔCt^ method [31]. All reactions were performed with three technical replicates (*n* = 3), and the experiments were repeated three times.

### 2.4. In Situ PCR

To investigate the tissue-specific expression of *PcNPF2.7* in *P. cornutum* roots, *in situ PcNPF2.7* mRNA levels in the root cross-sections were amplified as described previously [32]. Briefly, roots from 3-week-old seedlings were fixed overnight on ice in a solution containing 63% (*v*/*v*) ethanol, 5% (*v*/*v*) acetic acid, and 2% (*v*/*v*) formaldehyde. The fixed roots were embedded in 5% (*w*/*v*) agarose, and 50 µm-thick sections were prepared using a microtome (RM2245, Leica, Wetzlar, Germany). The sections were subjected to genomic DNA digestion using DNase I (TaKaRa, Dalian, China), followed by first-strand cDNA synthesis. Sections not subjected to reverse transcription (NO-RT) served as a negative control. PCR was performed to label the *PcNPF2.7* mRNA or *18S* RNA (positive control) in the root sections with digoxin. Gene-specific primers for *PcNPF2.7* (P11 and P12) and 18S RNA (P13 and P14) are listed in Table S1. Finally, the samples were stained using BM purple AP substrate (Sigma, Darmstadt, Germany) for 30 min, and the signal was observed using a fluorescence microscope (DM6B/DFC7000T, Leica, Wetzlar, Germany).

### 2.5. Transient Gene Expression in Tobacco

To determine the subcellular localization of PcNPF2.7, its full-length coding sequence was amplified from *P. cornutum* cDNA via PCR with Primers P15 and P16 (Table S1). Then, the *PcNPF2.7* fragment was fused into the plant expression vector p35S-eGFP carrying green fluorescent protein using the In-Fusion HD cloning kit (TaKaRa, Dalian, China) to obtain the recombinant vector p35S-PcNPF2.7-eGFP. Subsequently, the vectors p35S-eGFP and p35S-PcNPF2.7-eGFP were transformed into *Agrobacterium tumefaciens* (GV3101) via the heat shock method [14], and the bacterial solution was slowly injected into the tobacco (*Nicotiana benthamiana*) leaves according to the method described previously [33]. The green fluorescent protein (GFP) signal was observed using a confocal laser scanning microscope system (SP8 SR, Leica, Wetzlar, Germany) after infiltration for 3 days.

### 2.6. Functional Characterization of PcNPF2.7 in Arabidopsis

The effects of *PcNPF2.7* on plant growth, ion accumulation, and the expression of key genes involved in NO_3_^−^ and Na^+^ transport were investigated by ectopically transforming *PcNPF2.7* into Arabidopsis. As *PcNPF2.7* is predominantly expressed in the root stele of *P. cornutum* (as shown in the results), to avoid any potential effects on ion transport caused by non-targeted gene expression in other tissues, a root stelar-specific promoter in Arabidopsis (the promoter of *AtNPF2.3*, which is only expressed at the root stele and can maintain stable expression levels under NaCl treatment) was used to drive *PcNPF2.7* expression [28]. The *AtNPF2.3* (*pAtNPF2.3*) promoter was obtained using the primers P17 and P18 (Table S1). Then, the promoter sequence of *pAtNPF2.3*, followed by the full-length coding region of *PcNPF2.7*, was fused into the plant expression vector *pBIB-Basta* using the In-Fusion HD cloning kit with primer pairs P19 and P20 (Table S1). The recombinant vector was introduced into *A. tumefaciens* GV3101 and then transformed into wild-type Arabidopsis (Col-0) using the floral-dip method [34]. The relative expression level of *PcNPF2.7* in the roots and shoots of 3-week-old T_3_ homozygous transgenic lines was analyzed by qRT-PCR. Two transgenic lines (labeled as OE1 and OE2) were randomly selected for subsequent experiments.

To analyze the effects of stelar-specific expression of *PcNPF2.7* on the salt tolerance of Arabidopsis, the wild-type (WT) and transgenic lines (OE1 and OE2) were grown hydroponically following germination, as previously described [15]. Three-week-old seedlings were then treated with 1/2 Hoagland solution (control) or 1/2 Hoagland solution containing 25 or 75 mM NaCl, or 25 or 75 mM NaNO_3_. After being treated for 1 week, the dry weight of the shoots and roots were measured. The relative plasma membrane permeability and malondialdehyde (MDA) content in the leaves were measured as described previously [35,36]. Six biological replicates were performed for the abovementioned physiological parameters (*n* = 6).

The Cl^−^ content in oven-dried tissues was determined using a chloride analyzer (Model 926, Sherwood Scientific Ltd., Cambridge, UK) [30], and the NO_3_^−^ content was determined by the colorimetric method using a UV spectrophotometer (UV-2102 C, Unico Instrument Co., Ltd., Shanghai, China) as described previously [37]. The K^+^ and Na^+^ contents were determined using a flame spectrophotometer (Model 410 Flame; Sherwood Scientific, Ltd., Cambridge, UK), according to the method described previously [15]. Six biological replicates were performed for the abovementioned ion contents (*n* = 6).

### 2.7. Analysis of the Abundance of Na^+^ and NO_3_^−^ Transport-Related Genes

To analyze the effects of stelar-specific expression of *PcNPF2.7* on the expression of key genes involved in Na^+^ and NO_3_^−^ transport under salt stress, 3-week-old seedlings of WT, OE1, and OE2 grown in hydroponics were exposed to 1/2 Hoagland solution supplemented with 75 mM NaCl or 75 mM NaNO_3_ for 24 h. Then, the roots and shoots were immediately harvested, and the relative expression levels of the genes encoding Na^+^ transporters [*AtNHX1* (AT5G27150), *AtSOS1* (AT2G01980), and *AtHKT1;1* (AT3G62260)] and NO_3_^−^ transporters [*AtNPF2.3* (AT3G45680), *AtNRT1.5* (AT1G32450), and *AtCLCa* (AT5G40890)] were determined by qRT-PCR as described previously in “2.3”. *AtACTIN2* (AT3G18780) was used for internal standard, and the specific primers were listed in Table S1. All reactions were performed with three technical replicates (*n* = 3), and the experiments were repeated three times.

### 2.8. Data Analysis

All the data are presented as means with standard deviation, and data analysis was performed by analysis of variance using SPSS statistical software (v25.0, SPSS Inc., Chicago, IL, USA). Duncan’s multiple range tests were used to detect differences among means at a significance level of *p* < 0.05.

## 3. Results

### 3.1. Cloning and Characterization of PcNPF2.7

The full-length cDNA of *PcNPF2.7* is 1815 bp, comprising a 61-bp 5′ untranslated region, a 1686-bp open reading frame that encodes a protein consisting of 561 amino acid residues, and a 68-bp 3′ untranslated region (Figure S1). Phylogenetic analysis showed that, among the NAXT proteins in Arabidopsis, PcNPF2.7 exhibited a strong evolutionary relationship with AtNPF2.7 (Figure S2). The alignment revealed that PcNPF2.7 shared over 93% sequence identity with NPF2.7 proteins from Brassicaceae species, including *A. lyrata* subsp., *Brassica napus*, *Eutrema salsugineum*, and *Capsella rubella*, with all proteins containing 12 transmembrane domains (Figure S3).

### 3.2. The Expression Pattern of PcNPF2.7

**Figure 1:**
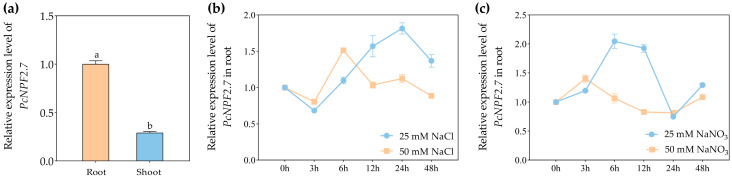
The expression analysis of *PcNPF2.7* in 4-week-old *P. cornutum*. (**a**) The expression level of *PcNPF2.7* in the root and shoot under control conditions. The expression level in the root was considered “1”. Different letters on the columns indicate significant differences (*p* < 0.05, *n* = 3). (**b**) The expression level of *PcNPF2.7* in the root under 25 mM or 50 mM NaCl treatment for 3, 6, 12, 24, and 48 h. (**c**) The expression level of *PcNPF2.7* in the root under 25 mM or 50 mM NaNO_3_ treatment for 3, 6, 12, 24, and 48 h. The expression level under 25 mM NaCl or NaNO_3_ at 0 h was considered “1” in (**b**,**c**), respectively. All experiments were repeated three times with similar results.

Under the control condition (no additional salts), the relative expression level of *PcNPF2.7* in *P. cornutum* roots was significantly higher than in shoots (Figure 1a). Subsequently, the relative *PcNPF2.7* levels in the roots of *P. cornutum* were analyzed after 3, 6, 12, 24, and 48 h of treatment with 25 and 50 mM NaCl. The results showed that the expression abundance of *PcNPF2.7* in the roots of *P. cornutum* first increased, peaking at 24 and 6 h, respectively, and then decreased under 25 and 50 mM NaCl treatment (Figure 1b). Furthermore, we analyzed the changes in the expression level of *PcNPF2.7* in the roots of *P. cornutum* after 25 and 50 mM NaNO_3_ treatment. The results showed that under 25 mM NaNO_3_ treatment, the expression abundance of *PcNPF2.7* in the roots of *P. cornutum* first increased, peaking at 6 h, and then decreased to the level before the treatment (Figure 1c). Under 50 mM NaNO_3_ treatment, the *PcNPF2.7* levels in the roots of *P. cornutum* increased at 3 h, then gradually decreased, and then tended to stabilize with the extension of the treatment time (Figure 1c). These results indicated that NaCl and NaNO_3_ treatment induced *PcNPF2.7* expression in the roots.

### 3.3. Tissue and Subcellular Localization of PcNPF2.7

**Figure 2:**
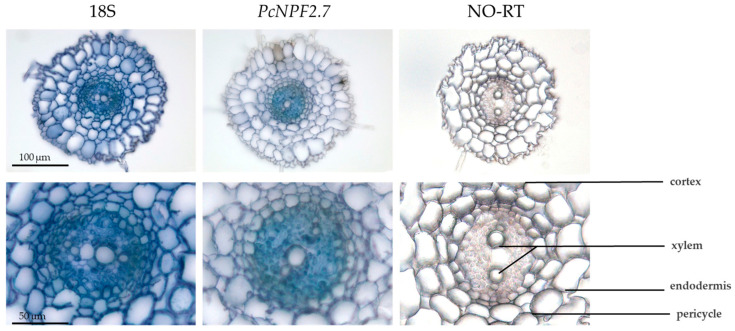
*In situ* PCR analysis of *PcNPF2.7* in the root of *P. cornutum*. 18S RNA is the positive control expressed in all cells. NO-RT denotes the negative control without reverse transcription. The blue signal indicates the gene expression location.

We analyzed the tissue localization of *PcNPF2.7* in the roots of *P. cornutum* using *in situ* PCR. In the positive (18S) and negative (NO-RT) controls, all and none of the root cells were stained blue, respectively. However, the transcript of *PcNPF2.7* was only stained blue in the root stele (Figure 2). This finding indicated that, unlike *AtNPF2.7*, which is primarily expressed in the cortical cells of the root of Arabidopsis [27], *PcNPF2.7* was specifically expressed in the stelar tissue of *P. cornutum* roots.

**Figure 3:**
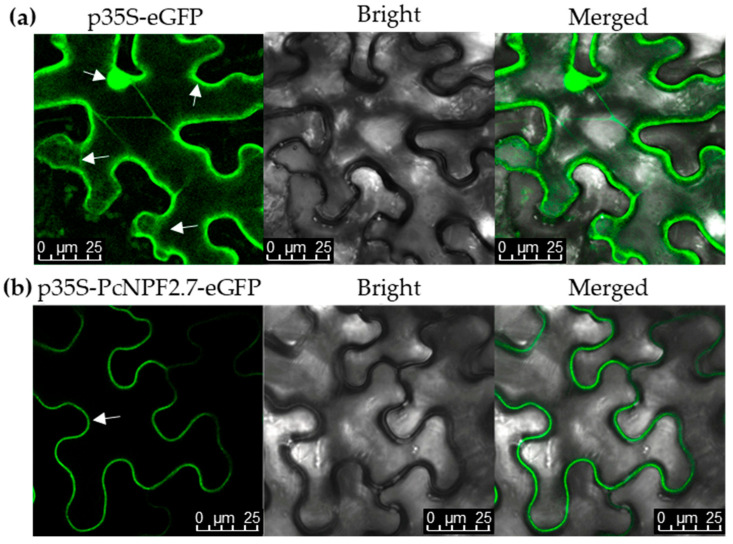
Subcellular location of PcNPF2.7 in leaf epidermal cells of tobacco. (**a**) The images of fluorescence signal elicited by the transient expression of empty vector. (**b**) The images of fluorescence signal elicited by the transient expression of *PcNPF2.7*, indicating that PcNPF2.7 was localized on the plasma membrane. The p35S-eGFP and p35S-PcNPF2.7-eGFP are the images of fluorescence signal elicited by the transient expression of empty vector and PcNPF2.7-eGFP fusion protein, respectively. Bright field is the image of leaf epidermal cell morphology under bright field condition. Merge is the image after fusing the fluorescence signal with bright field. The arrows show the fluorescence locations.

In order to determine the subcellular localization of PcNPF2.7, we constructed a GFP-PcNPF2.7 fusion protein and transiently expressed it in tobacco epidermal cells. As shown in Figure 3, the fluorescence of GFP was observed in the cytoplasm, plasma membrane, and nucleus of the leaf epidermal cells of tobacco that were transiently transformed with the empty vector (labeled p35S-eGFP). However, under transient expression of the PcNPF2.7-GFP fusion protein (labeled p35S-PcNPF2.7-eGFP), the green fluorescence signal was localized only on the plasma membrane of tobacco mesophyll cells (Figure 3), indicating that PcNPF2.7 was located on the plasma membrane.

### 3.4. Effects of PcNPF2.7 Expression on the Growth of Transgenic Lines of Arabidopsis Under NaCl Treatment

The effect of PcNPF2.7 on salt tolerance and ion accumulation was investigated by overexpressing it in Arabidopsis under the control of the *AtNPF2.3* promoter, which is only expressed at the root stele and could maintain stable expression levels under salt stress [37,38]. Two transgenic lines, OE1 and OE2, were randomly selected in the experiments (Figure S4).

**Figure 4:**
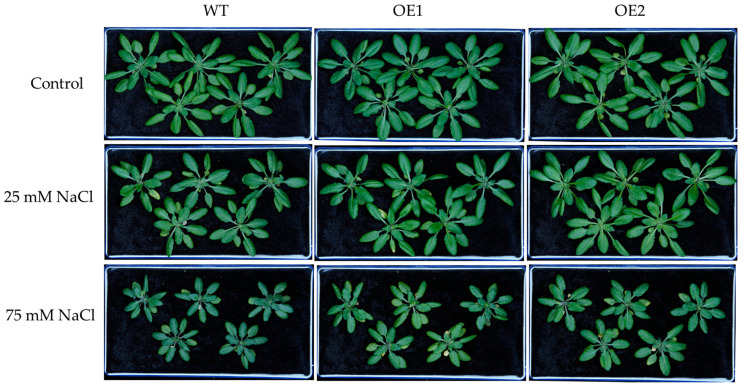
The growth phenotypes of 3-week-old WT and transgenic lines of Arabidopsis with stelar-specific overexpression of *PcNPF2.7* (OE1 and OE2) under 25 and 75 mM NaCl treatment for 7 d.

**Figure 5:**
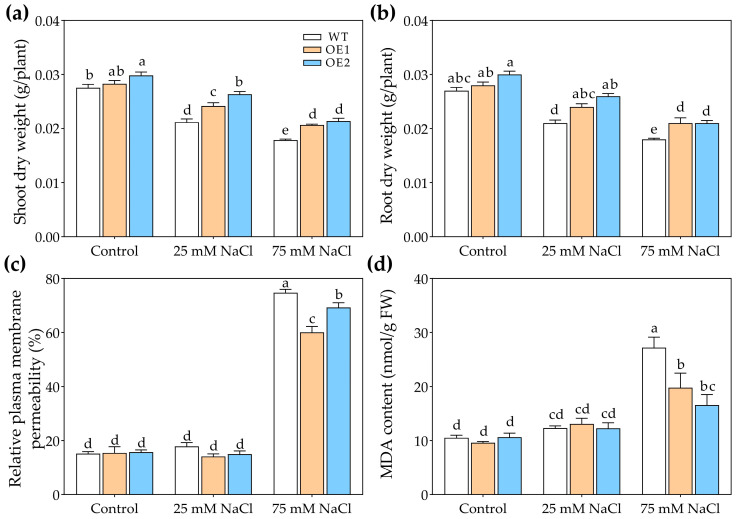
The growth parameters of 3-week-old WT and transgenic lines of Arabidopsis with the stelar-specific overexpression of *PcNPF2.7* (OE1 and OE2) under 25 mM and 75 mM NaCl for 7 d. (**a**) Shoot dry weight. (**b**) Root dry weight. (**c**) Relative plasma membrane permeability of the leaves. (**d**) MDA content in the leaves. Six biological replicates were performed for the abovementioned physiological parameters (*n* = 6). Different letters on the columns indicate significant differences (*p* < 0.05).

Under control conditions, there were no obvious differences in the growth of WT, OE1, and OE2 lines (Figure 4). Under 25 or 75 mM NaCl treatment, the transgenic lines exhibited better growth than WT (Figure 4). Moreover, under the control condition, there was no significant difference in the dry weight of WT, OE1, and OE2 lines (Figure 5a,b). Under 25 mM or 75 mM NaCl treatments, the transgenic lines (OE1 and OE2) exhibited higher shoot dry weight compared to WT (Figure 5a). And the root dry weight of OE1 and OE2 was significantly higher than that of WT under 75 mM NaCl treatment (Figure 5b).

In order to further determine the effects of *PcNPF2.7* expression on the salt tolerance of *A. thaliana*, we measured the relative plasma membrane permeability and malondialdehyde (MDA) content. The results indicated no significant differences in the relative plasma membrane permeability and MDA content of the leaves between the *PcNPF2.7* overexpression lines and the WT under control treatment and 25 mM NaCl treatment (Figure 5c,d). While under 75 mM NaCl treatment, the relative plasma membrane permeability and MDA content in the leaves of the *PcNPF2.7* overexpression lines were significantly lower than those of the WT (Figure 5c,d), indicating a lower degree of cell membrane damage in the *PcNPF2.7* overexpression lines under higher NaCl concentration.

These results indicated that the ectopic expression of *PcNPF2.7* enhanced the tolerance of *A. thaliana* to NaCl stress.

### 3.5. Effects of PcNPF2.7 Expression on the Cl^−^, NO_3_^−^, Na^+^, and K^+^ Ion Accumulation in Transgenic Lines of Arabidopsis Under NaCl Treatment

**Figure 6:**
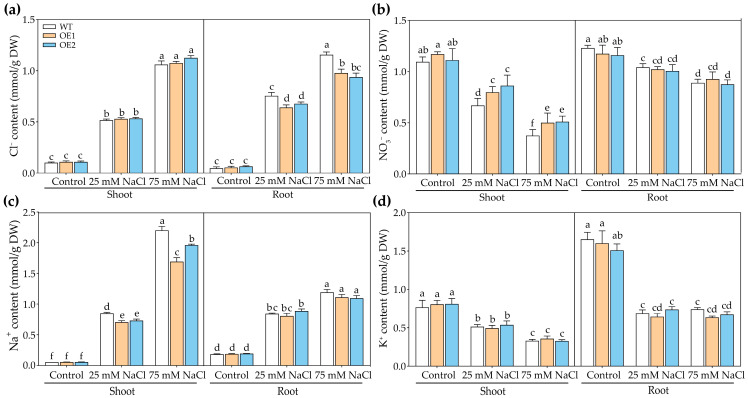
The ion concentration in 3-week-old WT and transgenic lines of Arabidopsis with stelar-specific overexpression of *PcNPF2.7* (OE1 and OE2) under 25 and 75 mM NaCl treatment for 7 d. (**a**) Cl^−^ content. (**b**) NO_3_^−^ content. (**c**) Na^+^ content. (**d**) K^+^ content. Six biological replicates were performed for the abovementioned ion contents (*n* = 6). Different letters on the columns indicate significant differences (*p* < 0.05).

Regardless of the control or NaCl treatments, no significant difference in the Cl^−^ content of the shoots was observed among OE1, OE2, and WT, whereas the Cl^−^ content in the roots of OE1 and OE2 was lower than that of the WT under NaCl treatments (Figure 6a). Under control conditions, no significant differences were found in the NO_3_^−^ content in the roots and shoots of OE1, OE2, and WT (Figure 6b). Under 25 or 75 mM NaCl treatments, the NO_3_^−^ content in the shoots of OE1 and OE2 was higher compared to the WT; although no significant difference was observed in the NO_3_^−^ content in the roots of WT, OE1, and OE2 (Figure 6b). These results indicated that the ectopic expression of *PcNPF2.7* increased NO_3_^−^ accumulation in the shoot but reduced Cl^−^ accumulation in the root under NaCl stress.

Under control conditions, there were no significant differences in the Na^+^ content in the roots or shoots of OE1, OE2, and WT (Figure 6c). Interestingly, under 25 or 75 mM NaCl treatments, the Na^+^ content in the shoots of OE1 and OE2 was lower compared to the WT (Figure 6c). Regardless of the control or NaCl treatment, no significant differences were observed in the K^+^ content in the roots or shoots of OE1, OE2, and WT (Figure 6d). These observations demonstrated that although the stelar-specific overexpression of *PcNPF2.7* did not affect K^+^ accumulation in plants, it reduced Na^+^ accumulation in the shoots.

### 3.6. Effects of PcNPF2.7 Expression on the Expression of Genes Related to NO_3_^−^ and Na^+^ Transport in Transgenic Lines of Arabidopsis Under NaCl Treatments

Next, we explored the reason underlying the elevation of NO_3_^−^ content induced by stelar-specific overexpression of *PcNPF2.7* in NaCl-treated shoots. We analyzed the expression levels of *AtNPF2.3* and *AtNRT1.5*/*AtNPF7.3*, which have been shown to mediate xylem loading of NO_3_^−^ [28,39] in roots of OE1, OE2, and WT under 75 mM NaCl treatment. In addition, we analyzed the expression of *AtCLCa*, which is involved in vacuolar NO_3_^−^ compartmentation [40], in the shoots. The results showed that the expression levels of *AtNPF2.3* and *AtNRT1.5* in the roots and *AtCLCa* in the shoots of OE1 and OE2 were comparable to those in WT under control conditions or NaCl treatments, (Figure S5). These findings indicated that the increase in NO_3_^−^ content in the shoots of OE1 and OE2 was directly associated with *PcNPF2.7* expression, but it was not associated with the expression of other key genes involved in xylem loading of NO_3_^−^ and vacuolar NO_3_^−^ compartmentation.

**Figure 7:**
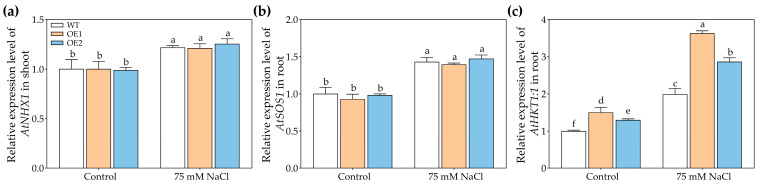
The expression level of key genes associated with Na^+^ transport in 3-week-old WT and transgenic lines of Arabidopsis with the stelar-specific overexpression of *PcNPF2.7* (OE1 and OE2) under 75 mM NaCl for 24 h. (**a**) The expression level of *AtNHX1* in shoot. (**b**) The expression level of *AtSOS1* in root. (**c**) The expression level of *AtHKT1;1* in root. Different letters on the columns indicate significant differences (*p* < 0.05, *n* = 3). All experiments were repeated three times with similar results.

We also analyzed the reason for the decrease in Na^+^ content mediated by *PcNPF2.7* overexpression in the shoots of NaCl-treated plants. For this purpose, we compared the expression of genes encoding Na^+^ transporters, including AtNHX1 (involved in vacuolar Na^+^ compartmentation) [41], AtSOS1 (mediates root Na^+^ efflux) [42], and AtHKT1.1 (involved in the unloading of Na^+^ from xylem to parenchyma cells) [43], in OE1, OE2, and WT. The results showed that regardless of control or NaCl treatment, the relative abundances of *AtNHX1* in the shoots and *AtSOS1* in the roots of OE1 and OE2 were comparable to those observed in the WT (Figure 7a,b). However, under 75 mM NaCl treatment, the *AtHKT1;1* level in the roots of OE1 and OE2 were significantly higher than that in WT (Figure 7c), indicating that the stelar-specific *PcNPF2.7* overexpression might be induced by the expression of *AtHKT1;1* in the roots, leading to more Na^+^ unloading from xylem to parenchyma cells, thus reducing Na^+^ transport to the shoots and decreasing Na^+^ levels in the shoots.

### 3.7. Effects of PcNPF2.7 Expression on the Growth of Transgenic Lines of Arabidopsis Under NaNO_3_ Treatments

**Figure 8:**
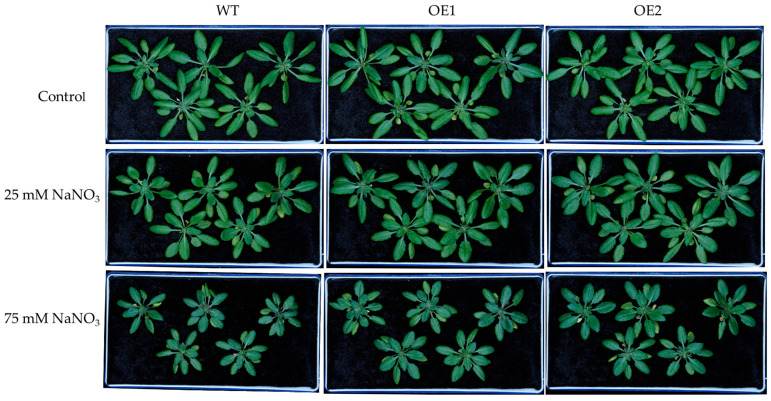
The growth phenotypes of 3-week-old WT and transgenic lines of Arabidopsis with the stelar-specific overexpression of *PcNPF2.7* (OE1 and OE2) under 25 mM and 75 mM NaNO_3_ for 7 d.

**Figure 9:**
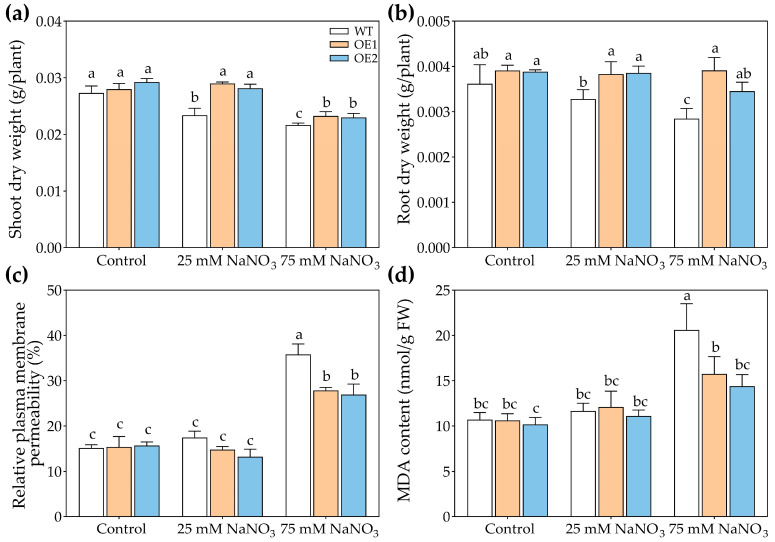
The growth parameters of 3-week-old WT and transgenic lines of Arabidopsis with the stelar-specific overexpression of *PcNPF2.7* (OE1 and OE2) under 25 mM and 75 mM NaNO_3_ for 7 d. (**a**) Shoot dry weight. (**b**) Root dry weight. (**c**) Relative plasma membrane permeability of the leaves. (**d**) MDA content in the leaves. Six biological replicates were performed for the abovementioned physiological parameters (*n* = 6). Different letters on the columns indicate significant differences (*p* < 0.05).

Since *PcNPF2.7* expression in *P. cornutum* roots was also induced by NaNO_3_ treatments, we speculated that *PcNPF2.7* might play a role in the response of plants to external NO_3_^−^ changes. Therefore, we compared the differences in the growth and ion accumulation among OE1, OE2, and WT under NaNO_3_ treatments. The results showed comparable growth of WT, OE1, and OE2 under control conditions (Figure 8). However, the transgenic lines exhibited better growth than WT under NaNO_3_ treatments (Figure 8). The shoot dry weight and the root dry weight of OE1 and OE2 were higher than those of WT under 25 mM or 75 mM NaNO_3_ treatments (Figure 9a,b), indicating that *PcNPF2.7* overexpression improved the resistance of *A. thaliana* to high concentrations of NaNO_3_. Under the control and 25 mM NaNO_3_ treatment, the relative plasma membrane permeability and MDA content in the leaves were comparable among all three lines (Figure 9c,d). Under 75 mM NaNO_3_ treatment, the transgenic lines exhibited significantly lower relative plasma membrane permeability and MDA content in the leaves compared to the WT (Figure 9c,d). Therefore, *PcNPF2.7* overexpression lines exhibited a lower degree of cell membrane damage under 75 mM NaNO_3_ treatment.

### 3.8. Effects of PcNPF2.7 Expression on the NO_3_^−^, Na^+^, and K^+^ Ion Accumulation and Salt Tolerance in Transgenic Lines of Arabidopsis Under NaNO_3_ Treatments

**Figure 10:**
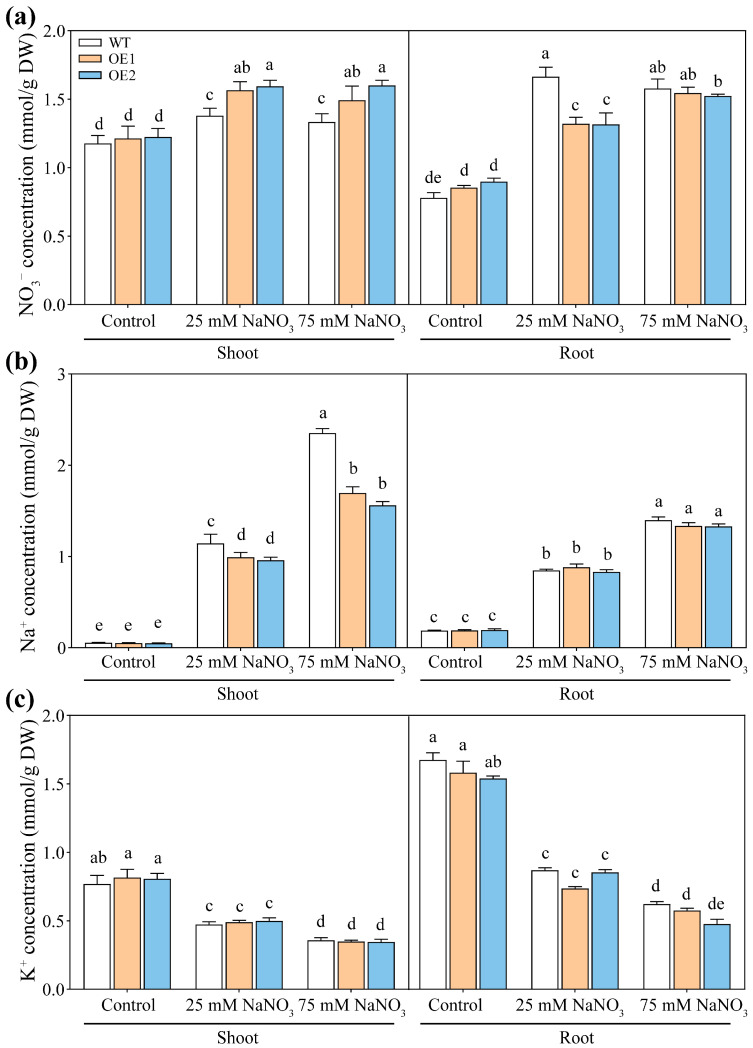
The ion concentration in 3-week-old WT and transgenic lines of Arabidopsis with the stelar-specific overexpression of *PcNPF2.7* (OE1 and OE2) under 25 mM and 75 mM NaNO_3_ for 7 d. (**a**) NO_3_^−^ content. (**b**) Na^+^ content. (**c**) K^+^ content. Six biological replicates were performed for the abovementioned ion contents (*n* = 6). Different letters on the columns indicate significant differences (*p* < 0.05).

Ion content analysis revealed that under control conditions, the NO_3_^−^, Na^+^, and K^+^ contents in the roots and shoots of *PcNPF2.7* overexpression lines were comparable to those of WT (Figure 10). However, under 25 mM NaNO_3_ treatment, NO_3_^−^ contents in the shoots and roots of transgenic lines were significantly higher and lower compared to WT, respectively (Figure 10a). Under 75 mM NaNO_3_ treatment, the shoots of OE1 and OE2 still exhibited higher NO_3_^−^ content compared to WT (Figure 10a). Under 25 mM and 75 mM NaNO_3_ treatment, Na^+^ content in the shoots of OE1 and OE2 was significantly lower (by 27% and 33%, respectively) compared to WT, with a more prominent decrease under higher NaNO_3_ concentration (Figure 10b). Taken together, the stelar-specific overexpression of *PcNPF2.7* increased NO_3_^−^ content and decreased Na^+^ content in the shoots of plants treated with higher NaNO_3_ concentration. In addition, the K^+^ contents in the roots and shoots were comparable among WT, OE1, and OE2 under either control or NaNO_3_ treatments (Figure 10c).

### 3.9. Effects of PcNPF2.7 Expression on the Expression of Genes Related to NO_3_^−^ and Na^+^ Transport Under NaNO_3_ Treatment

In order to investigate the effects of stelar-specific overexpression of *PcNPF2.7* on NO_3_^−^ and Na^+^ content under NaNO_3_ treatments, we performed qRT-PCR to analyze the levels of key genes involved in NO_3_^−^ and Na^+^ transport in transgenic lines. The results showed that under 75 mM NaNO_3_ treatment, the expressions of *AtNPF2.3* and *AtNRT1.5*/*AtNPF7.3* were comparable in the roots of all three lines (Figure S6a,b); however, the transgenic lines exhibited higher *AtCLCa* levels in the shoots compared to WT (Figure S6c). Furthermore, under 75 mM NaNO_3_ treatment, *AtNHX1* levels in the shoots and *AtSOS1* levels in the roots of OE1 and OE2 were comparable to those of WT (Figure S6d,e), and *AtHKT1;1* levels in the roots of OE1 and OE2 were significantly higher than that in WT (Figure S6f).

## 4. Discussion

The tissue-specific localization of genes encoding ion transport proteins or channels determines their functions in ion transport at the whole-plant level. Even when homologous proteins exhibit identical functions at the cellular level, their roles in ion transport within the plant can vary significantly due to the varied expression patterns of the encoding genes across different plant species [43,44]. For instance, the HKT homologous proteins in some plants mediate Na^+^ absorption at the cellular level [43]. However, in *A. thaliana*, the homologous gene *AtHKT1;1* is primarily expressed in the parenchyma cells around the xylem of the root, and the encoded protein mainly participates in mediating the unloading of Na^+^ from the xylem sap to the parenchyma cells [44]. While in rice and wheat, the *HKT2;1* homologous genes are predominantly expressed in the epidermal and cortical cells of the root, mediating the absorption of low Na^+^ concentration by the root from the external medium [44]. The in situ PCR analysis in the present study showed that *PcNPF2.7* is primarily expressed in the stelar cells of the root of *P. cornutum* (Figure 2), different from the tissue localization of *AtNPF2.7* in *A. thaliana* (mainly expressed in the cortical cells of the root) [27]. Thus, the function of PcNPF2.7 in the ion transport in *P. cornutum* root might vary from that of AtNPF2.7 in *A. thaliana*. Studies have shown that ion transport proteins or channels located in the xylem tissue of plant roots play a key role in regulating the ion transport and distribution in the roots and shoots of plants [45]. qRT-PCR analysis indicated that *PcNPF2.7* expression in the roots of *P. cornutum* was significantly induced by NaCl and NaNO_3_ (Figure 1b,c), and thus, we speculated that PcNPF2.7 might participate in the transport and distribution of ions between the root and shoots of *P. cornutum* under NaCl and NaNO_3_ treatments.

Studies in *A. thaliana* have found that among the NAXT family members, *AtNPF2.3* can mediate NO_3_^−^ efflux at the cellular level [28]. Further analysis revealed that *AtNPF2.3* is mainly expressed in the stelar cells of the roots, and the encoded protein is located in the plasma membrane [28]. Compared to the WT plants, salt-treated *atnpf2.3* mutant lines exhibited significantly decreased NO_3_^−^ content in the shoots, with significantly lower salt tolerance [28]. This finding indicated that *AtNPF2.3* participates in the long-distance transport of NO_3_^−^ from the roots to the shoots under salt stress, and plays an important role in the salt tolerance of *A. thaliana* [28]. In the current study, we found that, similar to AtNPF2.3, *PcNPF2.7* was located in the plasma membrane (Figure 3) and the coding gene was mainly expressed in the stelar cells of the root (Figure 2). Moreover, overexpressing *PcNPF2.7* driven by the root stelar cell-specific promoter in Arabidopsis under NaCl stress led to a significant increase in the NO_3_^−^ content in the shoots and overall salt tolerance of the plant (Figure 6b). These results indicated that the stelar-specific overexpression of *PcNPF2.7* might promote the long-distance transport of NO_3_^−^ from the roots to the shoots, potentially improving the salt tolerance of the plants. Studies have demonstrated that, in addition to *AtNPF2.3*, *AtNRT1.5*/*AtNPF7.3*, which is localized in the root xylem cells, also mediates the long-distance transport of NO_3_^−^ from the roots to the shoots of *A. thaliana* [39]. In the present study, under NaCl stress, the expression levels of *AtNPF2.3* and *AtNRT1.5* in the roots of *PcNPF2.7* overexpression lines were comparable to those in WT plants (Figure S5a,b). Meanwhile, the enhanced vacuolar NO_3_^−^ compartmentalization in the shoots contributed to NO_3_^−^ accumulation there [46]. Under NaCl stress, the expression level of *AtCLCa*, which is responsible for vacuolar NO_3_^−^ compartmentalization [40], in the shoots of *PcNPF2.7* overexpression lines was comparable to the levels in WT (Figure S5c). These results suggested that the increased NO_3_^−^ content in the shoots of *PcNPF2.7* overexpression lines under NaCl stress was directly triggered by the expression of *PcNPF2.7*, not by *AtNPF2.3* and *AtNRT1.5* in the roots or *AtCLCa* in the leaves. Taken together, these findings indicated that *PcNPF2.7* is directly involved in the long-distance transport of NO_3_^−^ from the roots to the shoots under salt stress.

Under higher NaNO_3_ stress, the NO_3_^−^ content in the shoots of *PcNPF2.7* overexpression lines was significantly higher than that in WT (Figure 10a). Additionally, the levels of *AtNPF2.3* and *AtNRT1.5* in the roots of *PcNPF2.7* overexpression lines were comparable to those in WT (Figure S6a,b), suggesting that *PcNPF2.7* also directly mediates the long-distance transport of NO_3_^−^ from the roots to the shoots under NaNO_3_ stress. However, excessive cellular accumulation of NO_3_^−^ can exert toxic effects on cytoplasmic metabolic activities. Interestingly, the *AtCLCa* levels in the shoots of *PcNPF2.7* overexpression lines were significantly higher than those in WT (Figure S6c), potentially facilitating the sequestration of excessive NO_3_^−^ from the cytoplasm into vacuoles in the shoots, thereby alleviating the toxic effects of excessive NO_3_^−^ in the leaves of *PcNPF2.7* overexpression lines.

In the present study, the stelar-specific overexpression of *PcNPF2.7* significantly reduced Na^+^ accumulation in the shoots of plants under both NaCl and NaNO3 stresses (Figure 6c and Figure 10b). These findings revealed another potential factor contributing to the enhanced salt tolerance observed in *PcNPF2.7* overexpression lines compared to WT. There is no evidence to suggest that NPF family proteins are directly involved in Na^+^ transport at the cellular level in plants. However, previous study found that Arabidopsis *AtNRT1.5*/*AtNPF7.3*, which mediates NO_3_^−^ transport from the roots to the shoots, can affect the Na^+^ transporter genes *AtNHX1* (involved vacuolar in Na^+^ compartmentation), *AtSOS1* (which mainly mediates Na^+^ efflux in the roots under high salt conditions), and *AtHKT1;1* (involved in the unloading of Na^+^ from xylem to parenchyma cells), thus indirectly affecting the accumulation of Na^+^ in plants [47]. Furthermore, mutations in the Cl^−^ channel gene *AtALMT9* can also affect the expression of *AtHKT1;1* and *CHX21*, potentially affecting Na^+^ accumulation in the shoots [48]. In the current study, under NaCl or NaNO_3_ stress, although no significant difference in *AtNHX1* levels in the shoots and *AtSOS1* levels in the roots was found between the *PcNPF2.7* overexpressing lines and WT (Figure 7a,b and Figure S6d,e), the *AtHKT1;1* levels in the roots were significantly higher in transgenic lines compared to WT (Figure 7c), which can help to unload Na^+^ from xylem to parenchyma cells, thereby reducing Na^+^ transport to the shoots and Na^+^ accumulation in the shoots [43]. However, the mechanisms underlying the influence of mutations or overexpression of NO_3_^−^ and other anion transporter genes on the expression of Na^+^ transporter genes still require further research.

## 5. Conclusions

This study demonstrated that *PcNPF2.7* was primarily expressed in the stele tissue of the roots of the xerophyte *P. cornutum*, and its expression levels in the roots were significantly induced by salt stress. *PcNPF2.7* overexpression in Arabidopsis driven by a stelar-specific promoter revealed that PcNPF2.7 facilitates the transport of NO_3_^−^ from the roots to the shoots, and modulates Na^+^ accumulation in the shoots by impacting the expression of *AtHKT1;1* in the roots. These results contributed to elucidating the salt tolerance mechanisms in the xerophyte *P. cornutum* and revealed potential candidate genes for improving salt tolerance in crops.

## Data Availability

Data is contained within the article or Supplementary Materials.

## Supplementary Materials

The following supporting information can be downloaded at: https://www.mdpi.com/article/10.3390/biology14111590/s1, Figure S1: The full-length nucleotide sequence and encoding amino acid sequence of *PcNPF2.7* from *P. cornutum*. Red marks are the start codon (ATG) and stop codon (TGA), respectively; Figure S2: Phylogenetic analysis of PcNPF2.7 with NAXT subclass members in *A. thaliana*. The identifiers for the AtNPF2.1-2.7 are At3G45720, At3G45690, At3G45680, At3G45700, At3G45710, At3g45660 and At3g45650.; Figure S3: The multiple alignment of amino acid sequence of PcNPF2.7 and NPF2.7 homologs from other plant species in the Brassicaceae. AtNPF2.7 (AT3G45650), AlNPF2.7 (Accession number in GenBank: CAH8267542.1), BnNPF2.7 (Accession number: WZY98940.1), CsNPF2.7 (Accession number: XP_010514891.1) and EsNPF2.7 (Accession number: XP_006419014.1) are the NPF2.7 homologs in *A. thaliana*, *A. lyrata* subsp, *Brassica napus*, *Eutrema salsugineum* and *Camelina sativa*, respectively. The red lines indicate the transmembrane domains of PcNPF2.7; Figure S4: RT-PCR analysis of PcNPF2.7 in transgenic lines of Arabidopsis with the stelar-specific overexpression of *PcNPF2.7* (OE1 and OE2); Figure S5: The expression level of key genes associated with NO_3_^−^ transport in 3-week-old WT and transgenic lines of Arabidopsis with the stelar-specific overexpression of *PcNPF2.7* (OE1 and OE2) under 75 mM NaCl for 24 h. (a) The expression level of *AtNPF2.3* in root. (b) The expression level of *AtNRT1.5* in root. (c) The expression level of *AtCLCa* in shoot. Different letters on the columns indicate significant differences (*p* < 0.05, *n* = 3). All experiments were repeated three times with similar results; Figure S6: The expression level of key genes associated with Na^+^ and NO_3_^−^ transport in 3-week-old WT and transgenic lines of Arabidopsis with the stelar-specific overexpression of *PcNPF2.7* (OE1 and OE2) under 75 mM NaNO_3_ for 24 h. Different letters on the columns indicate significant differences (*p* < 0.05, *n* = 3). All experiments were repeated three times with similar results; Table S1: Primers used in this study.
