# Supplementary material for: PcNPF2.7 from the Xerophyte Pugionium cornutum Facilitates Root-to-Shoot NO3− Transport and Affects Na+ Transport Under Salt Stress
Source: Biology (Basel). 2025 Nov 14;14(11):1590. doi: 10.3390/biology14111590 (PMC12650163; doi:10.3390/biology14111590)
Supplement: Supplementary file 1 [file biology-14-01590-s001.zip › biology-3891013-supplementary.pdf]

Supplementary Table S1 Primers used in this study

Primer	Sequences (5′-3′)
P1	TACCGCAGCGAAAATGAC
P2	CTGCCTTTCAGTAGCTGTTTTG
P3	GCTAATGTGAACCGTGTCCC
P4	CGAACCATGCGAAACTGCGTC
P5	TGAAGATGAAGGGATGTGGGC
P6	CTCTAACTTCAAAATCCCACCTGC
P7	CTTCCCCATGCTATCCTCCG
P8	TTCCCGTTCTGCCGTTGTG
P9	CGGTTCACATTAGCAACCGC
P10	CGCAGCGAAAATGACACGAA
P11	ATGTCTGTTTCAGTTTCTAGCGATG
P12	ATGTCTGTTTCAGTTTCTAGCGATG
P13	CAACCTGGTTGATCCTGCCAGT
P14	CTGATCCTTCTGCAGGTTCACCTAC
P15	GACTCTAGAAAGCTTATGGATGGTACTGTTTT
P16	ATTTAAATGTCGACAGATAGTGAAGGAGGG
P17	TGTTGGCCCAAGCTTTACCGTTGAAACGTTTTTTGTTCT
P18	GGGAAATTCGAGCTCTCGCATATAATTGGATCCGTTTTG
P19	TATATGCGAGAGCTCATGGATGGTACTGTTTTCGAAGAT
P20	GGGAAATTCGAGCTCTTAAGATAGTGAAGGAGGGACAGT
P*AtNHX1*-F	GGTGCTGTTGGGACTATTATTTC
P*AtNHX1*-R	CACAACACCCTCTCCGAATAC
P*AtSOS1*-F	CTACCCAATTTGTTCTACGC
P*AtSOS1*-R	TAGCTCCTCATCGTCTCCTA
P*AtHKT1;1*-F	TCTTCTTGGAGTGACGGTGC
P*AtHKT1;1*-R	TAGTTTCTCCGGTGTGTCGC
P*AtNPF2.3*-F	TGGAAACTAGGGTTTGGTCT
P*AtNPF2.3*-R	GGATTAGACTCGTGAAGGGA
P*AtNRT1.5*-F	TGTCATTGGACTTTCATCGC
P*AtNRT1.5*-R	CCATCATGGAATGTGAACCA
P*AtCLCa*-F	TCAGATGCCCAGAAGTGTTGTTCC
PAtCLCa-R	CCGTACAGATCCTTCCTGATATCC
PAtACTIN2-F	GAACCACACAGTGTTTTGTATAAGA
PAtACTIN2-R	CCATTACTGATACTTGGCTTGCT

```
1     AAGCAGTGGTATCAACGCAGAGTACATGGGGACAAAAAAGTGAAGGATTGATTTTCCAGATATGGATGGTACTGTTTTCGAAGATGCAGAAACGCTGATGTCAATAGATCACAGCACCAA
1                                                                    M  D  G  T  V  F  E  D  A  E  T  L  M  S  I  D  H  S  T  K
121   ACGTAAGGGTGGAGGTTGGATCACCTTTCCGTTTATGATAGCTACGTTGTTAGGCCTGACAATAGCAGCGTGGGGTTGGTTACTTAACTTGATCGTCTACCTGATTGAGGAATTCAACGT
41     R  K  G  G  G  W  I  T  F  P  F  M  I  A  T  L  L  G  L  T  I  A  A  W  G  W  L  L  N  L  I  V  Y  L  I  E  E  F  N  V
241   GAAGAGCATTGCAGCTGCTCAGATTGCCAACATTGTTAGTGGTTGTATCTGTATGGTTCCGGCCATTGGAGCCATTGCTGCTGACTCTTTCTTTGGAACCATCCCTGTTATCTCAGTGTC
81     K  S  I  A  A  A  Q  I  A  N  I  V  S  G  C  I  C  M  V  P  A  I  G  A  I  A  A  D  S  F  F  G  T  I  P  V  I  S  V  S
361   AGCTTTTATTTCTCTAATGGGCGTAGCTCTGCTGACTCTAACTGCTTCGCTTGACTCTCTAAGACCAAACTCCTGTGACACAGCTACAAGCTTTTGCCAGTCACCTTCAAAAATCCAGCT
121    A  F  I  S  L  M  G  V  A  L  L  T  L  T  A  S  L  D  S  L  R  P  N  S  C  D  T  A  T  S  F  C  Q  S  P  S  K  I  Q  L
481   AGCTGTCCTTTACACGGCCATAACTCTAGCCTCTATAGGAACAGGTGGGACACGGTTCACATTAGCAACCGCTGGTGCAAACCAGTACGAAAGGACTAAAGACCAAGGAAGCTTTTTCAA
161    A  V  L  Y  T  A  I  T  L  A  S  I  G  T  G  G  T  R  F  T  L  A  T  A  G  A  N  Q  Y  E  R  T  K  D  Q  G  S  F  F  N
601   CTGGTTTTTCTTCACAACGTATCTAGCTGCTGCTATAAGTGCAACCGCAATTGTCTACACCGAAGACAACATAAGCTGGACCCTCGGGTTTGGTCTGTGCATAGCTGCTAATCTCTTTAG
201    W  F  F  F  T  T  Y  L  A  A  A  I  S  A  T  A  I  V  Y  T  E  D  N  I  S  W  T  L  G  F  G  L  C  I  A  A  N  L  F  S
721   TTTTTGGTTTTCATTTCGGGGAAAAGATTCTACAAGCATGATAAACCCTTGGGAAGTCCTTTCACAAGTCTACTTCGTGTCATTTTCGCTGCGGTACTCAAAAGGAAAGCTGTGGTTTC
241    F  L  V  F  I  S  G  K  R  F  Y  K  H  D  K  P  L  G  S  P  F  T  S  L  L  R  V  I  F  A  A  V  L  K  R  K  A  V  V  S
841   AACTAACGAGAAAGACTATCACAATGAATCTACCGATACTCCCACAAAGAGTCTCAGGTTCTTTAACCGTGCAGCGTTGAAACAGGGCGACGAGGTTAAACCAGATGGCACAATTTGCAA
281    T  N  E  K  D  Y  H  N  E  S  T  D  T  P  T  K  S  L  R  F  F  N  R  A  A  L  K  Q  G  D  E  V  K  P  D  G  T  I  C  N
961   CCCTTGGAGACTATGCAGCGTTCAACAAGTGGAAGATTTCAAAGCAGTCATAAGAATCATCCCTCTTGCGTTAGCCACAATATTCTTGAGCACTCCAATAGCGATGCAGCTAAGCTTAAT
321    P  W  R  L  C  S  V  Q  Q  V  E  D  F  K  A  V  I  R  I  I  P  L  A  L  A  T  I  F  L  S  T  P  I  A  M  Q  L  S  L  I
1081  AGTTCTTCAAGGTCTGGTTATGGACAGGAGGCTTGGTCCTAACTTCAAAATCCCTGCAGGCTCTCTCCAAGTCATAACACTTGTTTCCACTTGCCTATTTATCATAGTGAACGATCGGTT
361    V  L  Q  G  L  V  M  D  R  R  L  G  P  N  F  K  I  P  A  G  S  L  Q  V  I  T  L  V  S  T  C  L  F  I  I  V  N  D  R  F
1201  TCTCTACCCATTTTACCAAAAGCTAACGGGAAAGTTCCTGACACCGCTCCAACGAGTCGGGATAGGCCATGTTTTCAACATTTTAAGCATGGCCGTGACTGCGATAGTTGAAGCAAAGAG
401    L  Y  P  F  Y  Q  K  L  T  G  K  F  L  T  P  L  Q  R  V  G  I  G  H  V  F  N  I  L  S  M  A  V  T  A  I  V  E  A  K  R
1321  ATTGAAGATTATACAAAACGGTCATCTTCTCGAATCATCATCTGTTGCTGACATGTCAGTCTTGTGGCTATTCCCTCCTCTAGTCATAGTAGGAATCGGAGAAGCCTTTCATTTCCCAGG
441    L  K  I  I  Q  N  G  H  L  L  E  S  S  S  V  A  D  M  S  V  L  W  L  F  P  P  L  V  I  V  G  I  G  E  A  F  H  F  P  G
1441  GAATGTAGCTCTGTGTTATCAAGAATTCCCAGAGTCTATGCGAAGCACAGCAACTTCGATAACCTCGGTGGTGATTGGGATATGTTTCTACACAAGCACTGCTTTGATCAATCTGATTCA
481    N  V  A  L  C  Y  Q  E  F  P  E  S  M  R  S  T  A  T  S  I  T  S  V  V  I  G  I  C  F  Y  T  S  T  A  L  I  N  L  I  Q
1561  GAGGACCACCGCGTGGTTACCAGATGACATAAACCATGGTAGAGTTGACAATGTGTACTGGGTTTTAGTCATAGGAGGAGTCTTGAATCTTGGCTATTTTCTTGTGTGTTCTTTGGTTTA
521    R  T  T  A  W  L  P  D  D  I  N  H  G  R  V  D  N  V  Y  W  V  L  V  I  G  G  V  L  N  L  G  Y  F  L  V  C  S  L  V  Y
1681  CAAATACAGAAACCTTGAGGACGATGATCAGGAACAAGATGCTACTGTCCCTCCTTCACTATCTTAAAAGAACATGTTCTTGCAAGTTTATTTCAGTATGCTTTTCGAAAAAAAAAAAA
561    K  Y  R  N  L  E  D  D  D  Q  E  Q  D  A  T  V  P  P  S  L  S  *
1801  AAAAAAAAAA
```

Figure S1. The full-length nucleotide sequence and encoding amino acid sequence of *PcNPF2.7* from *P. cornutum*. Red marks are the start codon (ATG) and stop codon (TGA), respectively.

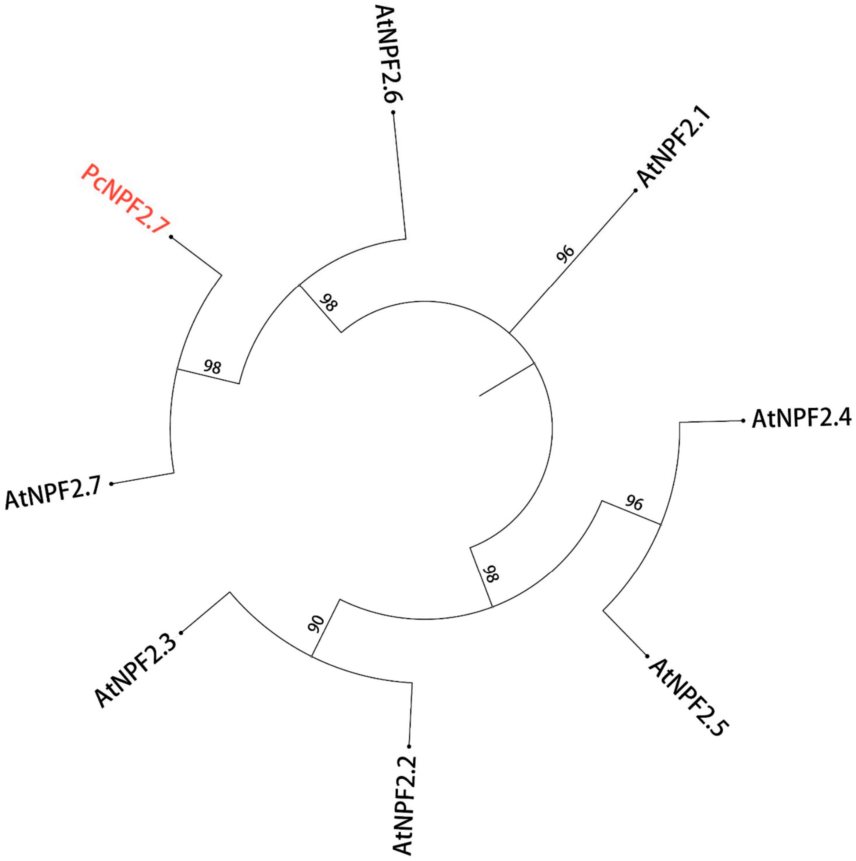

Figure S2. Phylogenetic analysis of PcNPF2.7with NAXT subclass members in *A. thaliana*. The identifiers for the AtNPF2.1-2.7 are At3G45720, At3G45690, At3G45680, At3G45700, At3G45710, At3g45660 and At3g45650.

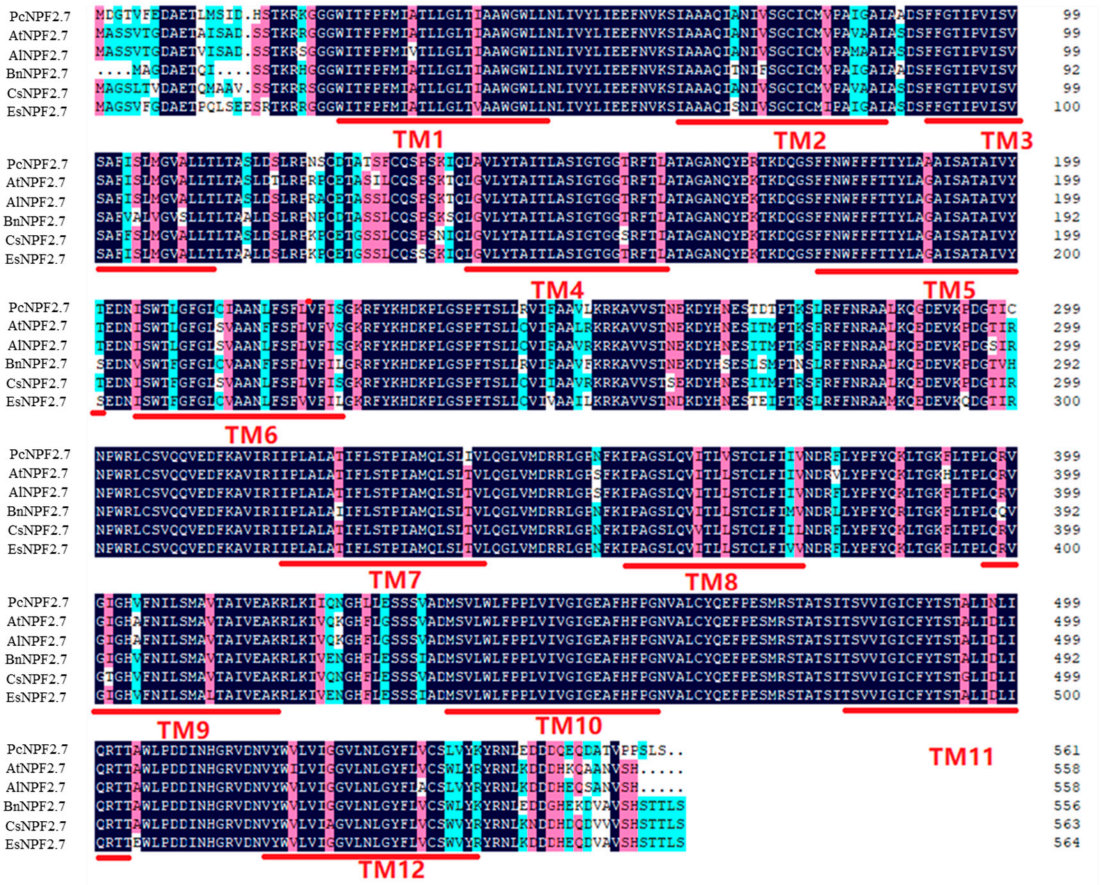

Figure S3. The multiple alignment of amino acid sequence of PcNPF2.7 and NPF2.7 homologs from other plant species in the Brassicaceae. AtNPF2.7 (AT3G45650), ALNPF2.7 (Accession number in GenBank: CAH8267542.1), BnNPF2.7 (Accession number: WZY98940.1), CsNPF2.7 (Accession number: XP_010514891.1) and EsNPF2.7 (Accession number: XP_006419014.1) are the NPF2.7 homologs in *A. thaliana*, *A.lyrata subsp*, *Brassica napus*, *Camelina sativa* and *Eutrema salsugineum*, respectively. The red lines indicate the transmembrane domains of PcNPF2.7.

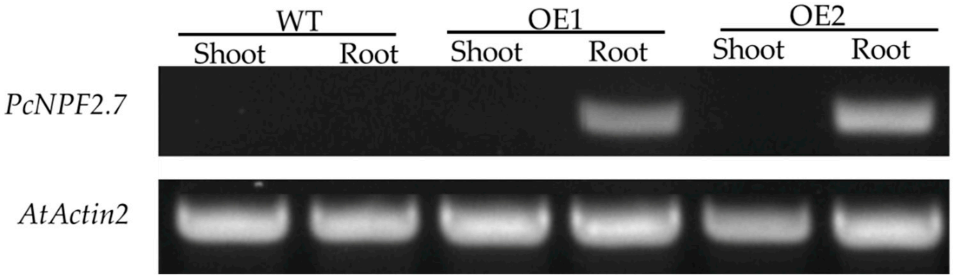

Figure S4. RT-PCR analysis of *PcNPF2.7* in transgenic lines of Arabidopsis with the stelar-specific overexpression of *PcNPF2.7* (OE1 and OE2).

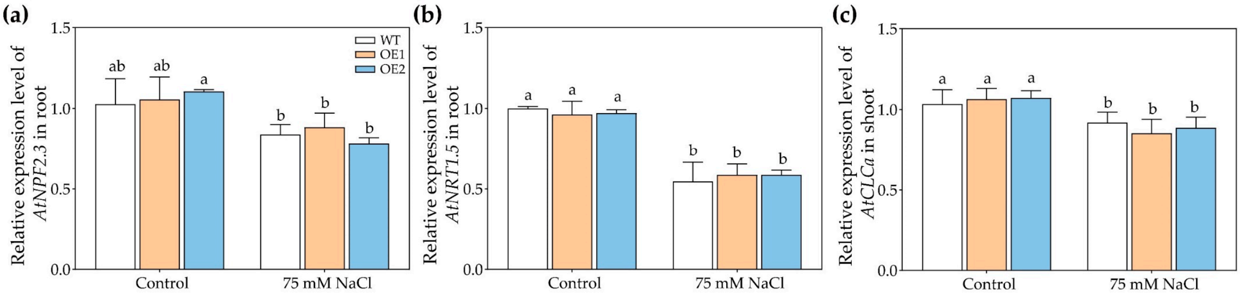

Figure S5. The expression level of key genes associated with NO_3^- transport in 3-week-old WT and transgenic lines of Arabidopsis with the stelar-specific overexpression of *PcNPF2.7* (OE1 and OE2) under 75 mM NaCl for 24 h. (a) The expression level of *AtNPF2.3* in root. (b) The expression level of *AtNRT1.5* in root. (c) The expression level of *AtCLCa* in shoot. Different letters on the columns indicate significant differences ($P < 0.05$, n=3). All experiments were repeated three times with similar results.

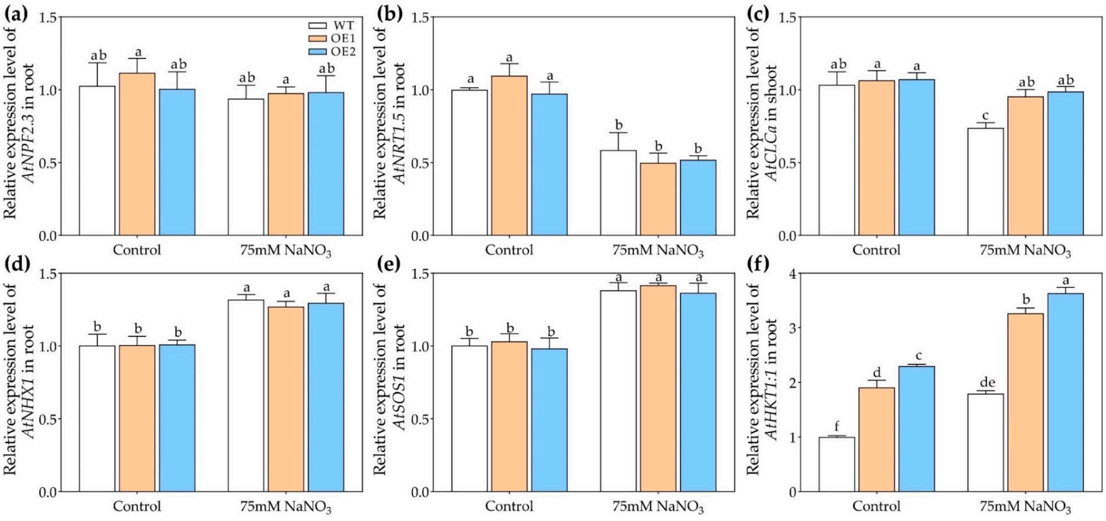

Figure S6. The expression level of key genes associated with Na^+ and NO_3^- transport in 3-week-old WT and transgenic lines of Arabidopsis with the stelar-specific overexpression of *PcNPF2.7* (OE1 and OE2) under 75 mM $NaNO_3$ for 24 h. Different letters on the columns indicate significant differences ($P < 0.05$, n=3). All experiments were repeated three times with similar results.
